# Diagnosis of Central Retinal Artery Occlusion in the Emergency Department Using POCUS: A Case Series

**DOI:** 10.24908/pocus.v6i2.14974

**Published:** 2021-11-23

**Authors:** Nicholas Cozzi, Kendall Stevens, Yeoshina Pillay, David Moore, Matthew Flannigan, Mariah Barnes, Matthew Singh, Melisa Gagrica, Christian Kolacki, Jennifer Bach, Dale McNinch, Drue Orwig, Jeffrey Jones

**Affiliations:** 1 Spectrum Health, Michigan State University College of Human Medicine Grand Rapids, MI

**Keywords:** POCUS, bedside ultrasonography, ocular ultrasound, central retinal artery occlusion

## Abstract

**Introduction: **Central Retinal Artery Occlusion is a cause of vision loss that warrants emergent evaluation. Ocular Point of Care Ultrasound (POCUS) is a non-invasive, inexpensive, and rapid modality to establish diagnosis with reduced time to consultation and treatment. **Methods: **This was a retrospective case series of patients evaluated at seven hospitals with diagnosis of CRAO over a two-year period. All patients underwent ocular POCUS performed by an emergency medicine clinician. **Results: **Nine patients were evaluated with mean vision loss of 21 hours. Overall, 88% of patients were diagnosed with CRAO, 75% possessing US confirmed retrobulbar spot sign (RBBS), and 38% confirmed diagnosis with fundoscopy.** Conclusion: **Ocular POCUS is an examination all emergency medicine clinicians should be able to perform. A rapid diagnosis of CRAO provides opportunity for vision improvement with initiation of treatment. The lack of guidelines for treatment of CRAO represents an opportunity for a multi-speciality collaboration to develop a diagnostic and treatment algorithm.

## Introduction

Painless vision loss represents 1.75% of annual emergency department (ED) visits^1^, ^2^ but can suggest not only an acute threat to vision, but also significant systemic pathology. The long term morbidity associated with vision impairment cannot be understated; it intercalates with every facet of a person's daily life which include professional and personal. Central Retinal Artery Occlusion, CRAO, is a common cause of painless vision loss that warrants prompt recognition and expert consultation. Thromboembolic disease related to cardiovascular or stroke risk factors 3, rheumatologic disease such as giant cell arteritis, or pathology localized to the eye can all present in a similar fashion 4. The Retrobulbar spot sign, or Hollenhorst sign/plaque was first described in 1992 consists of a bright echogenic white spot visualized in the central retinal artery in ocular POCUS and is consistent with cholesterol deposits reflective of thromboembolic disease 5, 6. 

For many causes of acute painless vision loss, dilated fundoscopic exam is considered the gold standard for diagnosis; however, this can be a challenging exam in some patients, especially in resource-limited settings without expert consultation available. It has previously been shown that point-of-care ultrasound (POCUS) can be used to characterize and evaluate both arterial and venous flow in cases of retinal vessel compromise using color Doppler imaging,^7^, ^8^ and to directly visualize retinal artery thrombus. Rapid diagnosis in the emergency department can decrease time to treatment, and increase the patient’s chances of regaining vision 4. 

## Methods

This study is an IRB-exempt case series of all patients seen at seven participating hospitals in West Michigan from July 2018 to July 2021 with a diagnosis of CRAO. All physicians have training and experience with POCUS as a means of rapid eye examination.These patients underwent POCUS imaging performed with prompt ophthalmology evaluation within 72 hours to be eligible for this study. Patient demographics, chief complaints, comorbidity, other radiographic studies, treatment in the ED, final disposition, and complications were obtained from the medical records using standardized abstraction forms. The emergency departments in all the participating hospitals are staffed by board-certified or board-eligible emergency physicians. A Zonare L10-5 linear array probe was to evaluate the eye in two perpendicular planes while the patient was supine with the head of the bed elevated thirty degrees. Ultrasound gel was used and slight pressure applied for visualization. Patient instructed to look side to side/up and down as this accentuates movement of retinal and vitreous pathology. Ultrasound exams were stored in QPath (a quality assurance and imaging documentation software) and used to confirm diagnostic accuracy through review by an emergency ultrasound fellowship trained physician. 

We were particularly interested in the feasibility and accuracy of EM providers using POCUS to diagnose acute CRAO. For the purposes of this study, feasibility will be defined as the ease of making a diagnosis and the accuracy will be based on image quality, retention of an adequate number of views to yield a diagnosis, and specificity when compared to a dilated fundoscopic exam by an ophthalmologist.

## Results

**Figure 1:**
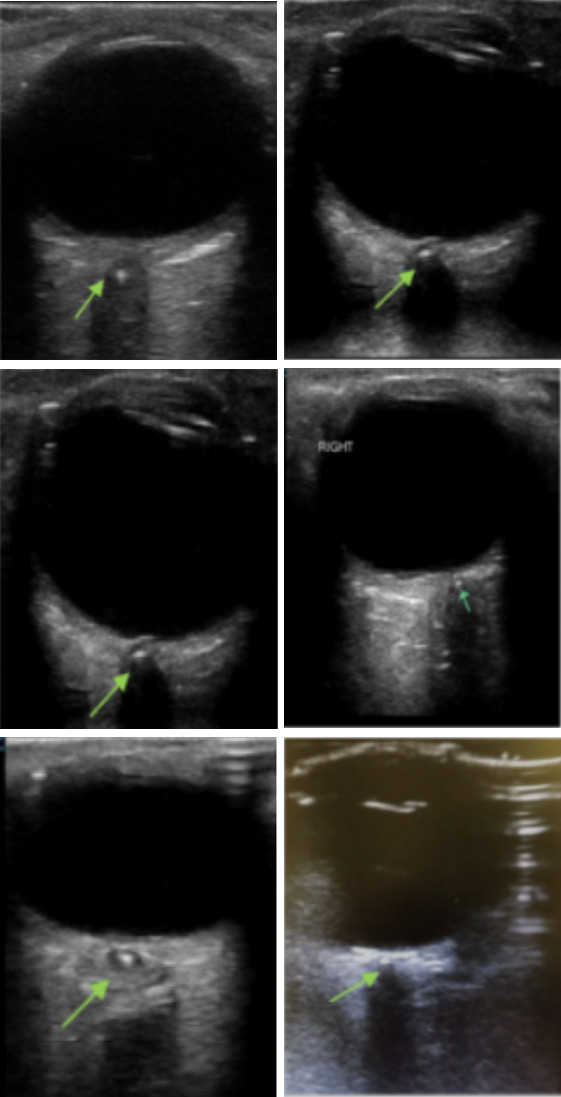
Retrobulbar Spot Sign (Hollenhorst sign/plaque). The above green arrow refers to six distinct patients with ocular-POCUS confirmed Retrobulbar spot sign (RBBS), also called the Hollenhorst sign/plaque, located in the central retinal artery.

Nine patients were seen at affiliated hospitals during the study period. The mean age was 64 +/- 19 SD; 55% were male. Overall, 88% of patients had risk factors for embolic occlusive disease, pre existing coronary arterial disease, peripheral vascular disease, carotid arterial disease, and/or atrial fibrillation. The average duration of monocular painless vision loss consisted of 21 hours when averaged over the 9 patients. However, when excluding two patients who had vision loss for greater than three days, the mean duration of vision loss for the remaining seven patients was 3 hours. All patients underwent ocular POCUS performed by an emergency medicine physician with 78% (7/9) patients having a POCUS confirmed, retrobulbar spot sign (RBBS; Figure 1, online Video S1). However, 38% of patients had ophthalmology confirmed diagnosis with fundoscopic examination. In terms of treatment, 67% of our case series underwent hyperbaric oxygen therapy (HBOT) with mean sessions being 3.6 at 2.8 atm. Of those patients undergoing HBOT, 40% had subjective improvement in their vision via Snellen Chart. One patient who was only able to recognize light and color improved where now this patient recognized hand motion. One patient underwent intra-arterial Tissue Plasminogen Activator (TPA) and notably, this was a teenager who did not recover vision.

## Discussion

Painless vision loss is a common emergency department complaint that is associated with high morbidity. Ocular POCUS is an inexpensive, non-invasive, and a rapid diagnostic modality emergency physicians can perform at the bedside to assess painless vision loss. Ocular POCUS is also useful for visualization of retinal detachment (RD), vitreous hemorrhage (VH), and posterior vitreous hemorrhage (PVH) which is displayed in table form. In addition, ocular ultrasound can assist in identifying optic nerve sheath edema due to increased intracranial pressure of greater than 20cm (sensitivity of 88%, specificity of 93%) 8. Ocular POCUS is a relatively new modality and one study comments on missed diagnosis of patients with CRAO due to providers not having the required training to perform this diagnostic maneuver. Retrobulbar spot sign is found in a subset of CRAO patients and is associated with a thromboembolic etiology. Our research indicates that RBBS may be sensitive in diagnosing CRAO, but not as specific given only 38% of our subset had CRAO confirmed via ophthalmology. An additional finding consistent with CRAO is evaluation of the arterial doppler signal to confirm dampened waveforms consistent with flow-limiting occlusion. The visualization of retrobulbar spot sign is associated with CRAO with two studies correlating the association to approximately 59% 5, 8, 9. Early visualization of RBBS (within 12 hours) and subsequent diagnosis of CRAO would reduce the time to diagnosis and HBOT. One study demonstrated that HBOT-treated non-arteritic CRAO patients were able to achieve an improvement of three lines on the Snellen visual acuity scale (38% vs. 17%, p=0.06,) 10, 11.

## Conclusion

Ocular POCUS is a non-invasive examination all emergency medicine clinicians should be able to perform. A rapid diagnosis of CRAO has the opportunity to reduce the time to treatment with the best chance for vision improvement. The lack of robust guidelines for the treatment of CRAO represents an opportunity for a multi-specialty collaboration to develop a structured diagnostic and treatment algorithm. 

## Statement of ethics approval/consent

All authors approved the version to be published and agreed to be accountable for all aspects of the work.

## Funding

The authors do not have any funding source. 

## Conflict of interest

The authors do not have any conflicts of interest to report.

## Supplementary Material

Video S1 Retrobulbar Spot Sign (Hollenhorst sign/plaque).
